# Optimising Suicide Risk Assessments for “Right-Siting” Psychiatric Presentations in the Accident & Emergency of a General Hospital in the City State of Singapore

**DOI:** 10.1192/bjo.2026.11414

**Published:** 2026-06-30

**Authors:** Palanivelu Sendhil Kumar, Su Yin Seow, Tan Ho Teck, Chao Tian

**Affiliations:** Sengkang General Hospital, Singapore, Singapore

## Abstract

**Aims:**

Sengkang General Hospital is one of the newest General Hospitals in Singapore. Among the other medical specialities, the department of psychiatry is one which is growing to provide the North East region of Singapore. Our close ally in managing psychiatric patients is the Institute of Mental Health which is a Tertiary Psychiatric care provider. The aim of the exercise was to equip the A&E with assessment and disposition of patients according to the needs and risks which the patient poses.

**Methods:**

The Psychiatry department working with the A&E and the Emergency Room of the Institute of Mental Health embarked to develop a protocol/workflow which would assist the A&E in assessing and equip them in placing the patients in the right setting based on the risks and needs. After many cycles of modelling and remodelling of the workflow a final version which was acceptable to all concerned was agreed upon.

This was important to develop as Sengkang General Hospital's department of psychiatry is still in the process of having a fully equipped unit. The junior manpower needed to cover the on call system is still a work in progress.This hasleftthe A&E devoid of full residential cover especially out of hours and during weekends.

Other issues which had to be simultaneously looked at were the comfort of the A&E staff who are at various levels of training, the ease of the Tertiary Care hospital (Institute of Mental Health) in accepting patients and also the comfort of patients to move between hospitals.

**Figure:**
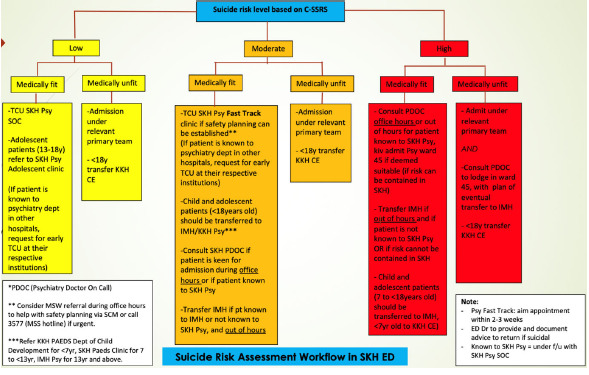

The assessments were broadly based on the CSSRS (Columbia-Suicide Severity Rating Scale). This scale is widely used in most of the hospitals in Singapore in suicide risk assessments. The workflow was colour coded for simplicity of use and understanding. Subtracts were developed for use in the child and Adolescent population. Another important aspect to to be taken into consideration was the “medical fitness” of the patient which would also be a deciding factor in the disposition.

**Results:**

A year now into the implementation: the results of the intervention have been promising.The erstwhile problem of difficulties in disposition of patients has reduced significantly, so that it has stopped being a “rolling agenda” item on the inter hospital meetings. There is a nice bonhomie between the 3 stakeholders in management of patients who present with suicide risk to the A&E. Patient movement between hospitals and departments is clearer. Patients get a clearer explanation on why they are moved to a particular destination.

**Conclusion:**

In a General Hospital setting, one of the most important aspect of service provision is the inter-departmental cooperation and exchange of expertise in management of patients. In this instance what seemed to be a simple intervention went a long way in managing a very important section of patients who present to the hospital at a time when theyare most needy and vulnerable. A clear assessment and management of these patients at a place where they are best managed was achieved with appropriate but simplistic intervention. A further scope to come up with similar interventions in psychiatric patients who are presenting with suicide risk is being discussed at the moment.

